# Amorphous Co-Mo-B Film: A High-Active Electrocatalyst for Hydrogen Generation in Alkaline Seawater

**DOI:** 10.3390/molecules27217617

**Published:** 2022-11-06

**Authors:** Xiaodong Fang, Xiangguo Wang, Ling Ouyang, Longcheng Zhang, Shengjun Sun, Yimei Liang, Yongsong Luo, Dongdong Zheng, Tairan Kang, Qian Liu, Feng Huo, Xuping Sun

**Affiliations:** 1 School of Mechanical Engineering, Chengdu University, Chengdu 610106, China; 2 Analytical Testing Center, School of Chemistry and Chemical Engineering, Institute of Micro & Nano Intelligent Sensing, Neijiang Normal University, Neijiang 641100, China; 3 Institute of Fundamental and Frontier Sciences, University of Electronic Science and Technology of China, Chengdu 610054, China; 4 School of Food and Biological Engineering, Chengdu University, Chengdu 610106, China; 5 Institute for Advanced Study, Chengdu University, Chengdu 610106, China

**Keywords:** amorphous Co-Mo-B film, alkaline seawater electrolysis, hydrogen evolution reaction

## Abstract

The development of efficient electrochemical seawater splitting catalysts for large-scale hydrogen production is of great importance. In this work, we report an amorphous Co-Mo-B film on Ni foam (Co-Mo-B/NF) via a facile one-step electrodeposition process. Such amorphous Co-Mo-B/NF possesses superior activity with a small overpotential of 199 mV at 100 mA cm^−2^ for a hydrogen evolution reaction in alkaline seawater. Notably, Co-Mo-B/NF also maintains excellent stability for at least 24 h under alkaline seawater electrolysis.

## 1. Introduction

The growing global energy crisis and environmental problems caused by the massive consumption of traditional non-renewable resources drive humans to explore eco-friendly and renewable energy, and hydrogen (H_2_) is a desirable alternative to fossil fuels on account of its high calorific value and pollution-free features [1,2,3,4]. Water electrolysis technology provides a facile and sustainable route for the massive generation of high-purity H_2_ [5,6,7,8,9,10,11,12,13,14,15]. Nevertheless, large-scale water electrolysis would doubtlessly exacerbate the shortage of freshwater. In contrast, the proportion of seawater is ~96.5% of the Earth’s water supply, making it have huge potential for large-scale H_2_ generation [16,17,18,19,20]. For hydrogen evolution reaction (HER), Pt-based materials are the most efficient catalysts, but the rareness and high cost heavily obstruct their practical applications. Meanwhile, on account of the complicated constituents and high salinity of seawater, most electrocatalysts would suffer from electrode corrosion [21,22]. Consequently, the exploitation of inexpensive, high-activity, and stable electrocatalysts for HER are urgently imperative.

Among the inexpensive alternatives, amorphous transitional metal-based materials exhibit considerable catalytic activity for HER, which could be attributed to the unique reverse electron transfer property and abundant coordinative unsaturated sites [23,24,25,26,27]. Notably, Co has been demonstrated as a catalytically active center in many Co-based HER electrocatalysts [28,29,30]. Recent studies also manifest that amorphous Co-B catalysts can efficiently catalyze the HER [28,31,32]. Furthermore, various optimization strategies, including using conducting supports, designing nanostructure, and incorporating other metals, have been employed to further improve HER performance [33,34,35]. Among these strategies, the inclusion of a second metal into monometallic catalysts can tune the intrinsic catalytic activity via optimizing the electronic structure [35,36].

Here, an amorphous Co-Mo-B film on Ni foam (Co-Mo-B/NF) was fabricated through a simple one-step electrodeposition process for HER in alkaline seawater. It suggests that amorphous Co-Mo-B/NF can afford a current density (*j*) of 100 mA cm^−2^ at an overpotential of 199 mV and a Tafel slope of 141 mV dec^−1^ in alkaline seawater. Furthermore, it also exhibits splendid durability during 24 h continuous electrolysis in alkaline seawater. This study not only offers an efficient catalyst for hydrogen production in alkaline seawater, but is also valuable for the design of amorphous transition metals and alloy electrocatalysts.

## 2. Results and Discussion

**Figure 1:**
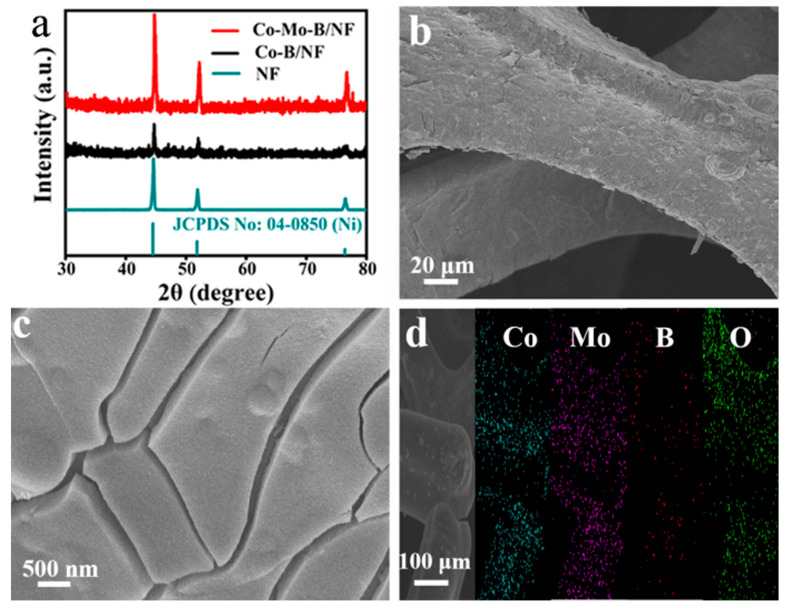
(**a**) XRD patterns of Co-Mo-B/NF, Co-B/NF, and NF. (**b**) Low- and (**c**) high-magnification SEM images of Co-Mo-B/NF. (**d**) SEM image and corresponding EDX elemental mapping images of Co-Mo-B/NF.

The Co-Mo-B/NF electrode was prepared via a one-step electrodeposition process at room temperature. The XRD patterns of Co-Mo-B/NF, Co-B/NF, and NF are depicted in Figure 1a. Both samples show three characteristic peaks located at 44.6°, 51.9°, and 76.6° originating from metallic NF (JCPDS No. 04-0850). Moreover, XRD patterns of Co-Mo-B/NF, Co-B/NF, and NF are similar, confirming the amorphous feature of Co-Mo-B and Co-B. The SEM images reveal the full coverage of NF substrate (Figure S1) by Co-Mo-B film (Figure 1b,c). As for Co-B/NF, the relevant SEM images are shown in Figure S2. The energy-dispersive X-ray spectroscopy (EDX) spectrum (Figure S3) and relevant elemental mapping images (Figure 1d) show that Co-Mo-B/NF is composed of Co, Mo, B, and O elements with a uniform distribution in the film.

**Figure 2:**
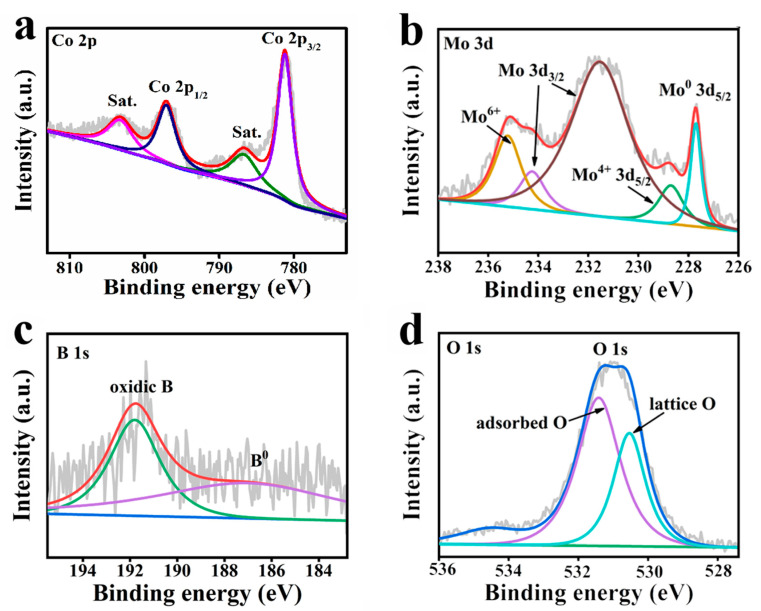
The refined XPS survey for Co-Mo-B/NF in (**a**) Co 2p, (**b**) Mo 3d, (**c**) B 1s, and (**d**) O 1s regions.

The XPS measurement spectrum (Figure S4) identifies that Co-Mo-B/NF consists of Co, Mo, B, and O elements, which is in accordance with the EDX data. The Co 2p spectrum (Figure 2a) shows that the characteristic peaks of 781.3 eV and 797.2 eV are well consistent with Co 2p_3/2_ and Co 2p_1/2_ of Co^2+^ [37]. Meanwhile, the binding energies of 786.6 and 803.4 eV are associated with satellite peaks (identified as Sat.). The Mo 3d spectrum of Co-Mo-B/NF in Figure 2b can be deconvoluted to 227.7 eV for Mo^0^ 3d_5/2_. The peaks at 228.7 and 231.53 eV are well consistent with 3d_5/2_ and 3d_3/2_ of Mo^4+^, while two peaks at 234.24 and 235.3 eV are attributed to 3d_3/2_ of Mo^6+^ [34,38,39]. In terms of B 1s spectrum (Figure 2c), characteristic peaks at 187 and 191.8 eV are assigned to metallic and oxidized boron, respectively [34]. Figure 2d shows the O 1s region and the peaks at 530.5 and 531.6 eV that are attributed to the lattice O and adsorbed O, respectively [40,41].

**Figure 3:**
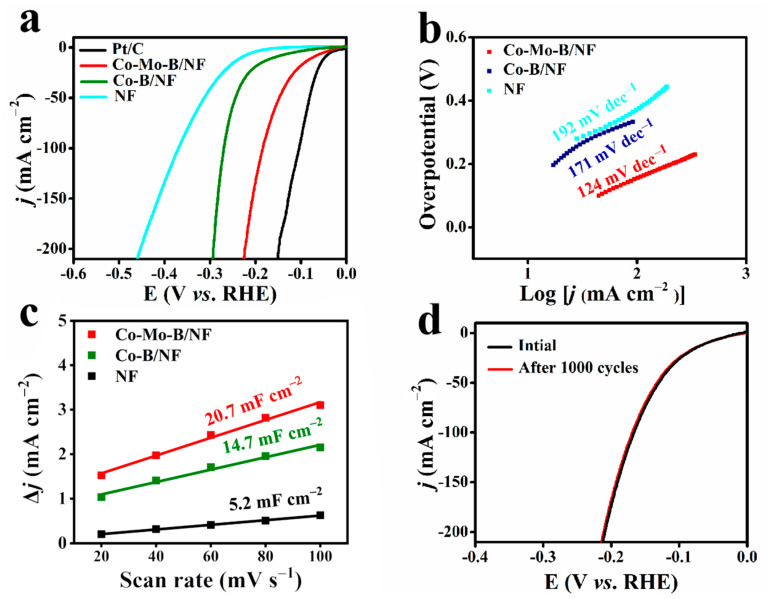
(**a**) Polarization curves, (**b**) Tafel plots for different materials in 1 M KOH. (**c**) Corresponding electrochemical double layer capacitances of Co-Mo-B/NF, Co-B/NF, and bare NF. (**d**) Polarization curves for Co-Mo-B/NF before and after 1000 CV cycles in 1 M KOH.

The electrocatalytic activity of different working electrodes toward the HER was firstly assessed by linear sweep voltammetry (LSV) curves (iR-corrected) in alkaline freshwater (1 M KOH). As observed in Figure 3a, bare NF has poor HER activity with a large overpotential, while the commercial Pt/C (20 wt.%) on NF (Pt/C loading of 2.6 mg cm^−2^) shows excellent HER performance with a low overpotential of 94 mV at 100 mA cm^−2^. Noticeably, to achieve the same *j* of 100 mA·cm^−2^, the Co-Mo-B/NF catalyst just needs an overpotential of 174 mV, superior to Co-B/NF counterpart (274 mV). Of note, our Co-Mo-B/NF compares favorably to the behaviors of most reported non-Pt HER electrocatalysts (Table S1). Subsequently, the Tafel slopes of catalysts were further determined to compare the reaction kinetics. As offered in Figure 3b, the Co-Mo-B/NF catalyst possesses a smaller Tafel slope (124 mV dec^−1^), in comparison with Co-B/NF (171 mV dec^−1^) and pure NF (192 mV dec^−1^), displaying superior catalytic kinetics on the Co-Mo-B/NF electrode during the HER process. Thus, Mo acts as an important role in enhancing the HER activity of Co-B/NF [41].

In order to further investigate the origin of the better activity of Co-Mo-B/NF, the double-layer capacitance (C_dl_) values were calculated from the cyclic voltammetry (CV) tests (Figure S6). As presented in Figure 3c, the calculated results display that the C_dl_ of Co-Mo-B/NF (20.7 mF cm^−2^) is larger than Co-B/NF (14.7 mF cm^−2^) and bare NF (5.2 mF cm^−2^). The larger C_dl_ of Co-Mo-B/NF facilitates the exposure of more active surface sites, thus, improving the electrocatalytic performance [42,43]. Furthermore, the stability of the Co-Mo-B/NF catalyst was also estimated based on continuous CV tests (Figure 3d). After 1000 cycles in 1 M KOH, the LSV curve for Co-Mo-B/NF shows a negligible change in *j* compared with the original one, implying that the Co-Mo-B/NF catalyst possesses remarkable stability.

**Figure 4:**
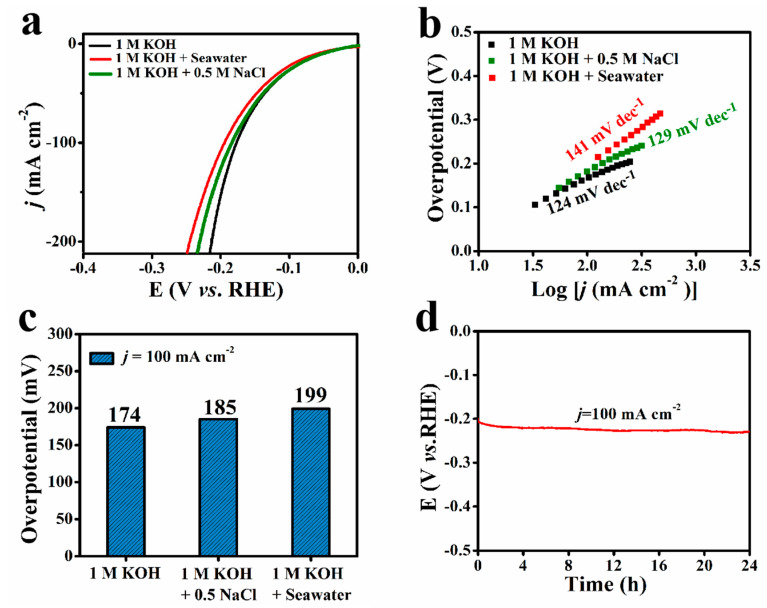
(**a**) Polarization curves of Co-Mo-B/NF in different electrolytes. (**b**) Overpotentials at 100 mA cm^–2^ for Co-Mo-B/NF in different electrolytes. (**c**) Tafel plots of Co-Mo-B/NF in different electrolytes. (**d**) Durability test of Co-Mo-B/NF in 1 M KOH + seawater.

To demonstrate the outstanding HER catalytic activity, the Co-Mo-B/NF was also explored in simulated seawater (1 M KOH + 0.5 M NaCl) and alkaline seawater (1 M KOH + seawater). The LSV curves (Figure 4a) show that the HER performance of Co-Mo-B/NF is still well maintained with a slight decay in simulated seawater compared to alkaline freshwater. As seen in Figure 4b, to obtain the *j* of 100 mA cm^−2^, Co-Mo-B/NF demands a low overpotential of 185 mV in simulated seawater, which is close to the value of alkaline freshwater (174 mV). After switching alkaline freshwater with alkaline natural seawater, the Co-Mo-B/NF catalyst exhibits an obvious activity decline by the reason of highly complex compositions of natural seawater. The ions or microorganisms in seawater can block some surface-active sites, resulting in lower catalytic activity of HER than in alkaline freshwater. Even so, the Co-Mo-B/NF catalyst still performs efficiently for catalyzing the HER in alkaline seawater. It can afford a *j* of 100 mA cm^−2^ at an overpotential of 199 mV. Remarkably, the Co-Mo-B/NF catalyst is superior in catalytic performance to many reported noble-metal-free HER electrocatalysts in alkaline seawater (listed in Table S2). Additionally, the Tafel slopes of Co-Mo-B/NF in different electrolytes were also determined based on the LSV curves. As illustrated in Figure 4c, the slope value in alkaline-simulated seawater (129 mV dec^−1^) is nearly close to alkaline freshwater (124 mV dec^−1^), while the slope value in alkaline seawater (141 mV dec^−1^) is marginally larger. Furthermore, the stability of the Co-Mo-B/NF catalyst was also assessed by the continuous electrolysis of 24 h in alkaline seawater. As displayed in Figure 4d, the Co-Mo-B/NF catalyst exhibits strong electrochemical stability at *j* of 100 mA·cm^−2^ without obvious decay after 24 h of operation. Importantly, as presented in Figure S8, the high-resolution XPS spectra of Co 2p, Mo 3d, B 1s, and O 1s for Co-Mo-B/NF after the stability test in alkaline seawater are similar to the initial analysis, demonstrating the highly durable performance of the Co-Mo-B/NF. The hydrogen produced by the Co-Mo-B/NF-driven electrolysis of alkaline seawater was collected by the drainage method. As shown in Figure S9, the Faraday efficiency for HER is calculated to be close to 100%, indicating that the amorphous Co-Mo-B/NF is important for the large-scale application of hydrogen production from seawater.

## 3. Experimental Section

### 3.1. Materials

Sodium molybdate dihydrate (Na_2_MoO_4_·2H_2_O, ≥99%), cobalt chloride hexahydrate (CoCl_2_·6H_2_O, A.R.), and citric acid (C_6_H_7_O_8_·H_2_O, A.R.) were obtained from Aladdin Industrial Co (Shanghai, China). Sodium borate (Na_2_B_4_O_7_·10H_2_O, A.R.) was bought from Tianjin Chemical Corporation (China). Pt/C (20 wt.% Pt) was provided by Alfa Aesar (China) Chemicals Co. Ltd. Ni foam (thickness: 1.6 mm, porosity: ~95%) was bought from KunShan (China) GuangJiaYuan New materials Co. Ltd. Seawater was taken from the coast of Bohai Sea in China.

### 3.2. Preparation of Co-Mo-B/NF and Co-B/NF

Firstly, CoCl_2_·6H_2_O (4.76 g), Na_2_MoO_4_·2H_2_O (1.46 g), C_6_H_8_O_7_·H_2_O (4.20 g), and Na_2_B_4_O_7_·10H_2_O (9.54 g) were dissolved in 100 mL of ultrapure water and kept mild, stirring for 15 min. The pretreated Ni foam (1.5 cm × 1 cm) was vertically immersed into the aqueous solution. After that, the Co-Mo-B film deposition was performed in a three-electrode setup. The Pt plate and saturated calomel electrode were used separately from the counter electrode and the reference electrode. Ni foam was served as the working electrode for the deposition process under −1.1 V for 60 min. For comparison, Co-B/NF was also synthesized by the same methods without Na_2_MoO_4_·2H_2_O.

### 3.3. Characterizations

X-ray diffraction (XRD) data were collected on Shimazu XRD-6100 X-ray diffractometer. Scanning electron microscopy (SEM) images were taken on GeminiSEM 300 microscope. The Thermo ESCALAB 250Xi spectrometer system was used to record the X-ray photoelectron spectra (XPS) data of the samples.

## 4. Conclusions

In conclusion, an amorphous Co-Mo-B film was electrodeposited on NF for hydrogen evolution electrocatalysis in alkaline seawater. The Co-Mo-B/NF catalyst requires a small overpotential of 199 mV to obtain a *j* of 100 mA·cm^−2^ and a small Tafel slope of 141 mV dec^−1^ in alkaline seawater. Moreover, it also exhibits high stability, as confirmed by 24 h of continuous electrolysis. This work is important because it can boost the development of bimetal-boron film materials for highly active HER electrocatalysts in the electrochemical seawater splitting process.

## Data Availability

Not applicable.

## Supplementary Materials

The following supporting information can be downloaded at: https://www.mdpi.com/article/10.3390/molecules27217617/s1, refs. [44,45,46,47,48,49,50,51,52,53,54,55,56] are cited in this file. Figure S1: (a) Low- and (b) high-magnification SEM images of bare NF; Figure S2: (a) Low- and (b) high-magnification SEM images of Co-B/NF; Figure S3: EDX spectrum of Co-Mo-B/NF; Figure S4: XPS survey spectrum of Co-Mo-B/NF; Figure S5: LSV curves of Co-Mo-B/NF, Co-B/NF, Pt/C, and bare NF for HER in 1 M KOH with a scan rate of 5 mV s^–1^ (without IR correction); Figure S6: CV curves for Co-Mo-B/NF (a), Co-B/NF (b), and bare NF (c) in the non-Faradaic capacitance current range at scan rates of 20, 40, 60, 80, and 100 mV s^–1^ in 1 M KOH; Figure S7: LSV curves of Co-Mo-B/NF in 1 M KOH, 1 M KOH + 0.5 M NaCl, and 1 M KOH + seawater with a scan rate of 5 mV s^–1^ (without IR correction); Figure S8: High-resolution XPS spectra of (a) Co 2p, (b) Mo 3d, (c) B 1s, and (d) O 1s regions for Co-Mo-B/NF after stability test in alkaline seawater; Figure S9: The Faradaic efficiency of Co-Mo-B/NF at 100 mA cm^–2^ in alkaline seawater; Table S1: Comparison of HER performance of Co-Mo-B/NF with recent reported electrocatalysts in alkaline freshwater; Table S2: Comparison of HER performance of Co-Mo-B/NF with recent reported electrocatalysts in alkaline seawater.
