# Supplementary material for: Amorphous Co-Mo-B Film: A High-Active Electrocatalyst for Hydrogen Generation in Alkaline Seawater
Source: Molecules. 2022 Nov 6;27(21):7617. doi: 10.3390/molecules27217617 (PMC9657096; doi:10.3390/molecules27217617)
Supplement: Supplementary file 1 [file molecules-27-07617-s001.zip › molecules-1977976-supplementary.pdf]

Supplementary Material

Amorphous Co-Mo-B film: a high-active electrocatalyst for hydrogen generation in alkaline seawater

Electrochemical tests:

All electrochemical experiments were performed with a CHI 760E electrochemical workstation in alkaline media, using Co-Mo-B/NF and Co-B/NF as working electrodes, graphite rod as counter electrode and Hg/HgO electrode as reference electrode, respectively. All measured potentials were referenced to that of reversible hydrogen electrode (RHE) in accordance with the Nernst equation: E (RHE) = E (Hg/HgO) + (0.098 + 0.059 × pH). To estimate the electrochemically active surface areas, the double-layer capacitance of the Co-Mo-B/NF, Co-B/NF, and bare NF were measured by conducting cyclic voltammetry (CV) at non-Faradaic potentials ranging from 0.0904 to 0.1904 V *vs.* RHE for HER at different scan rates of 20, 40, 60, 80, and 100 mV s^{-1} in 1 M KOH.

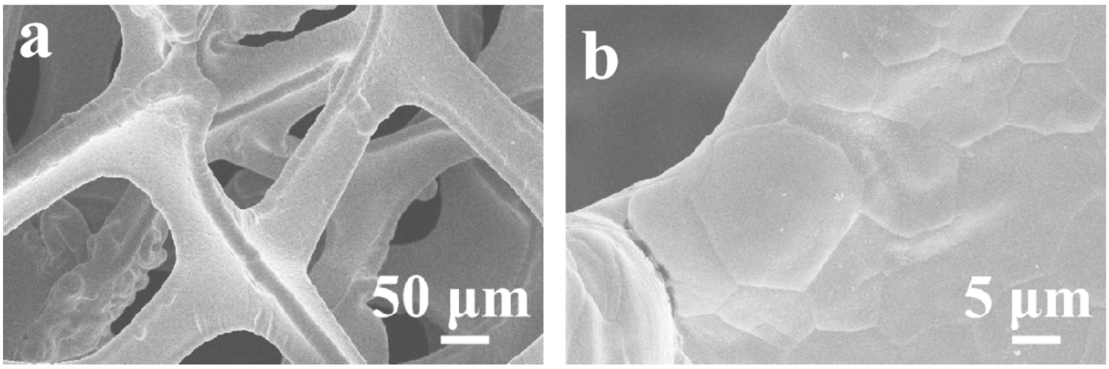

Figure S1. (a) Low- and (b) high-magnification SEM images of bare NF.

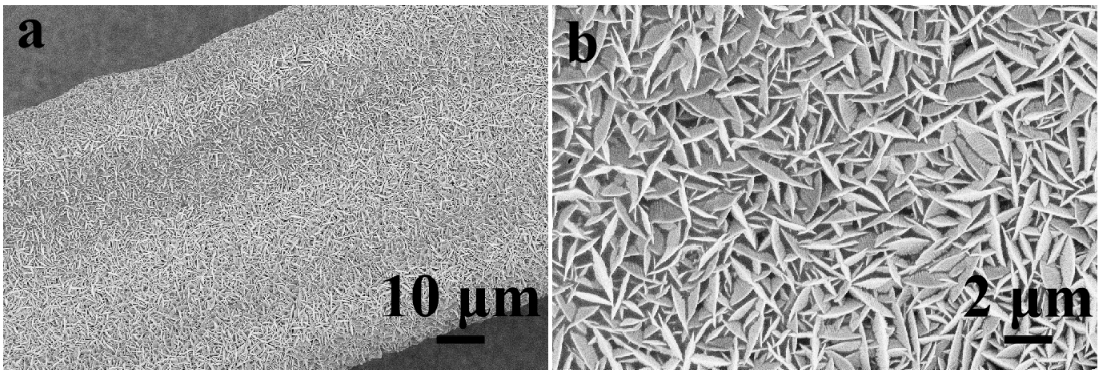

Figure S2. (a) Low- and (b) high-magnification SEM images of Co-B/NF.

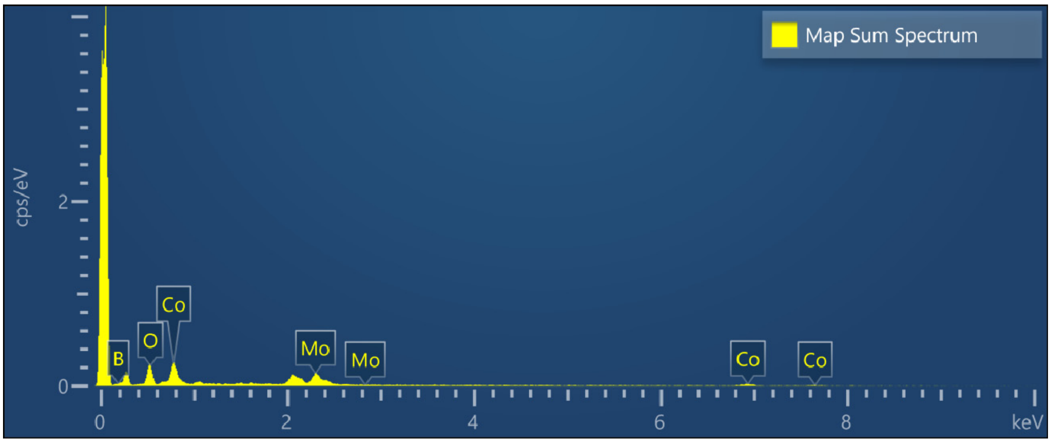

Figure S3. EDX spectrum of Co-Mo-B/NF.

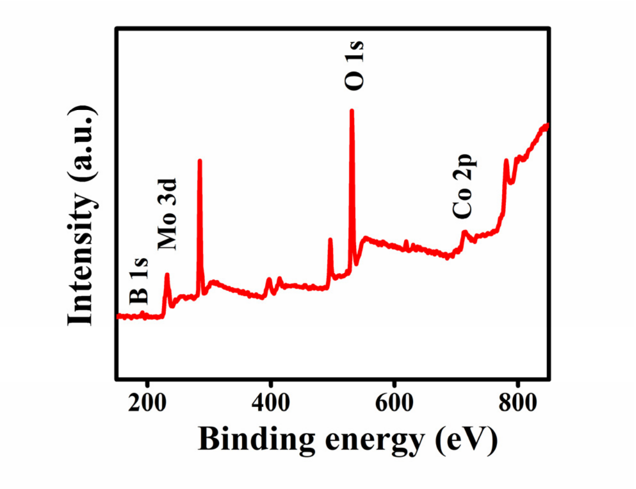

Figure S4. XPS survey spectrum of Co-Mo-B/NF.

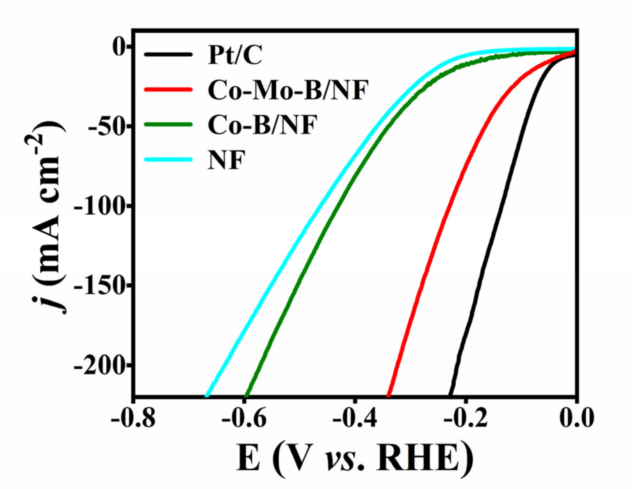

Figure S5. LSV curves of Co-Mo-B/NF, Co-B/NF, Pt/C, and bare NF for HER in 1 M KOH with a scan rate of 5 mV s^{-1} (without IR correction).

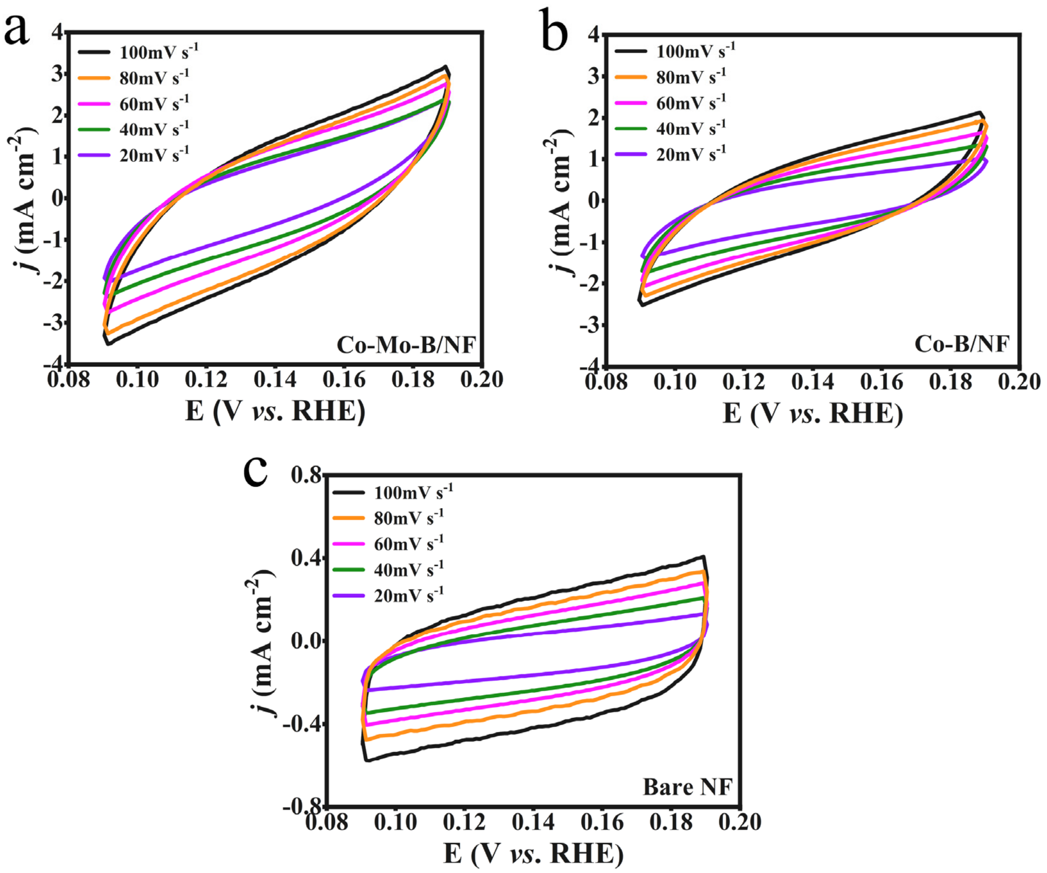

Figure S6. CV curves for Co-Mo-B/NF (a), Co-B/NF (b), and bare NF (c) in the non-Faradaic capacitance current range at scan rates of 20, 40, 60, 80, and 100 mV s^{-1} in 1 M KOH.

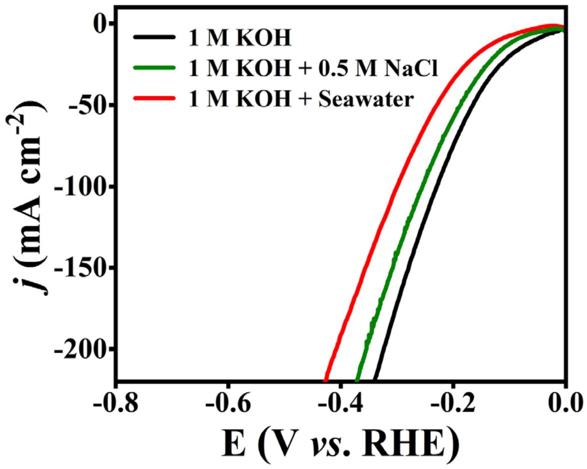

Figure S7. LSV curves of Co-Mo-B/NF in 1 M KOH, 1 M KOH + 0.5 M NaCl, and 1 M KOH + seawater with a scan rate of 5 mV s^{-1} (without IR correction).

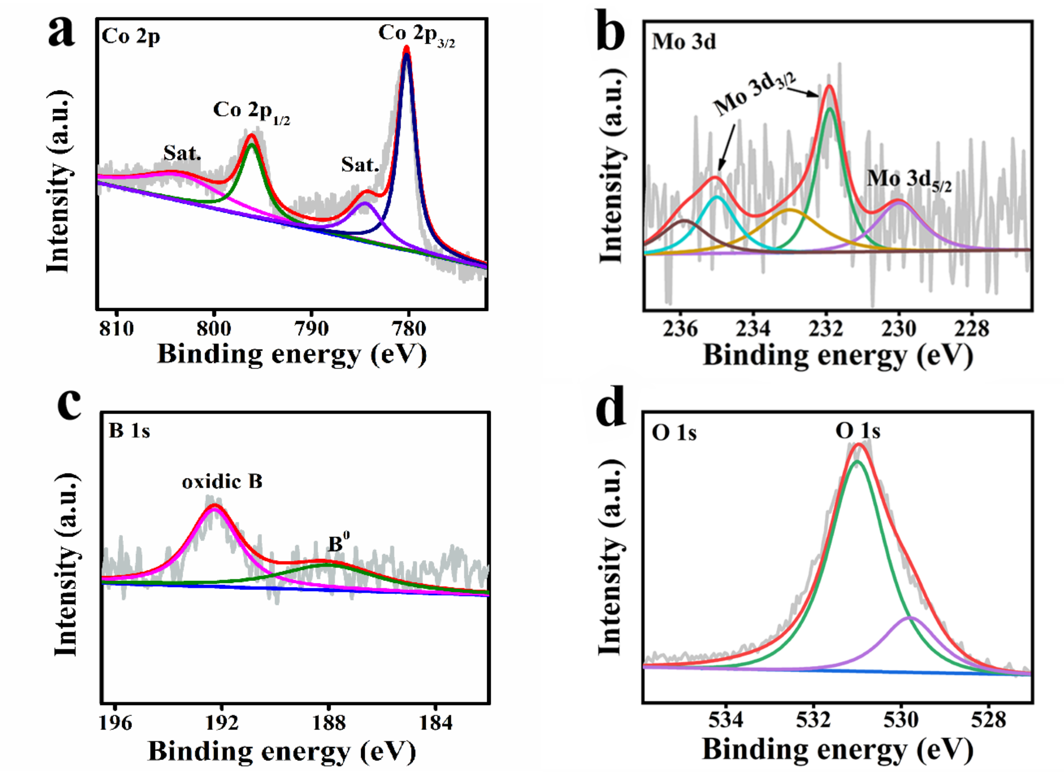

Figure S8. High-resolution XPS spectra of (a) Co 2p, (b) Mo 3d, (c) B 1s, and (d) O 1s regions for Co-Mo-B/NF after stability test in alkaline seawater.

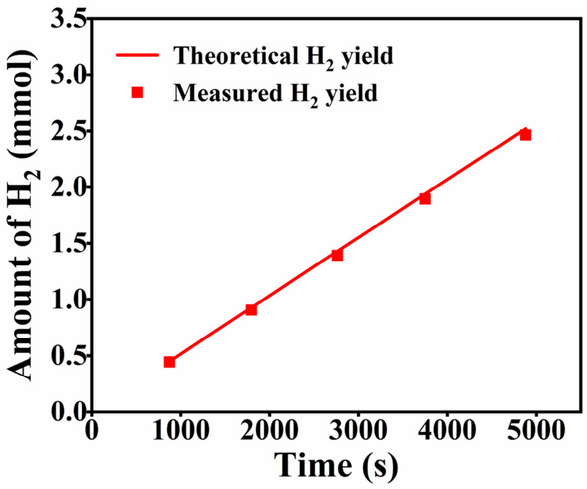

Figure S9. The Faradaic efficiency of Co-Mo-B/NF at 100 mA cm^{-2} in alkaline seawater.

Table S1. Comparison of HER performance of Co-Mo-B/NF with recent reported electrocatalysts in alkaline freshwater.

HER catalysts	Current density (mA cm^{-2})	Overpotential (mV)	Electrolyte	Ref.
Co-Mo-B/NF	100	174	1 M KOH	This work
Mo-CoP_x/NF	100	~300	1 M KOH	*Mater. Today Nano* **2022**, *18*, 100216.
$CoMoS_4$/β-$Co(OH)_2$	100	313	1 M KOH	*Nanoscale* **2016**, *8*, 18887–18892.
$NiCo_2O_4$	100	245	1 M NaOH	*Angew. Chem. Int. Ed.* **2016**, *55*, 6290–6294.
MoS_2-CoS_2@PCMT	100	300	1 M KOH	*J. Power Sources* **2021**, *514*, 230580.
HC-MoS_2/Mo_2C	100	354	1 M KOH	*Nat. Commun.* **2020**, *11*, 3724.
Co/CoMoN/NF	100	173	1 M KOH	*Adv. Sci.* **2022**, *9*, 2105313.
Ni_3S_2/MoS_2-CC	100	173	1 M KOH	*J. Mater. Chem. A* **2019**, *7*, 2895–2900.
CoN/NF	100	212	1 M KOH	*J. Mater. Chem. A* **2019**, *7*, 19728–19732.

Table S2. Comparison of HER performance of Co-Mo-B/NF with recent reported electrocatalysts in alkaline seawater.

**HER catalysts**	**Current density (mA cm^{-2})**	**Overpotential (mV)**	**Electrolyte**	**Ref.**
Co-Mo-B/NF	100	199	1 M KOH + seawater	This work
S-$NiMoO_4$@NiFe-LDH/NF	100	220	1 M KOH + seawater	*J. Colloid Interface Sci.* **2022**,*613*, 349–358.
CoCuS	100	289	1 M KOH + seawater	*Int. J. Hydrogen Energy* **2021**, *9*, 0360–3199.
Co-N,P-HCS	100	287	1 M KOH + seawater	*Adv. Mater.* **2022**, e2204021.
Ni_2P-Fe_2P/NF	100	252	1 M KOH + seawater	*Adv. Funct. Mater.* **2020**, *31*, 12006484.
NiCoHPi@Ni_3N/NF	100	182	1 M KOH + seawater	*ACS Appl. Mater. Inter.* **2022**, *14*, 22061–22070.
